# Awareness of DCD from the perspective of stakeholders in the Dutch school system: a qualitative study

**DOI:** 10.3389/fnhum.2025.1593912

**Published:** 2025-06-23

**Authors:** Femke van Abswoude, Meike Bakker, Linda Hanegraaf, Hidde Bekhuis

**Affiliations:** Behavioural Science Institute, Radboud University, Nijmegen, Netherlands

**Keywords:** developmental coordination disorder, awareness, interviews, teachers, students, support

## Abstract

**Introduction:**

Developmental Coordination Disorder (DCD) is one of the most prevalent developmental disorders, but largely unknown by educational professionals. The overall aim of this study was to explore awareness of DCD and ways to improve this among stakeholders in the Dutch primary school system.

**Methods:**

One semi-structured interviews were performed with teachers (including classroom teachers (*N* = 8) and physical education (PE) teachers (*N* = 2) at primary schools), students [from teachers’ college for primary education and/or physical education (*N* = 7)], and other stakeholders in the primary school organization [including school organization directors (*N* = 2) and special needs coordinators (*N* = 2)]. Thematic analysis was conducted.

**Results:**

Overall, 28.6% of the participants were aware of DCD, with percentages varying largely between participants from the school organization (100%), college students (14.3%), and teachers (10%). All participants endorsed the importance of raising awareness of DCD within the school system. It was recognized that participants can provide social support for children with DCD by stimulating both personal and social resources. All participants were open to ways to improve awareness, the responsibility of which is mostly ascribed to special need coordinators within schools.

**Discussion and conclusion:**

The results highlight both the lack of awareness of DCD among stakeholders in the primary school system in the Netherlands, as well as the importance of changing this. When teachers are aware of DCD, they can provide social support to the child which is essential to diminish the possible physical and psychological consequences. Increasing awareness within the Dutch school system may lead to better understanding, earlier diagnosis and earlier interventions for children with DCD.

## 1 Introduction

Developmental Coordination Disorder (DCD) is one of the most prevalent developmental disorders, affecting 5–7% of all children (Blank et al., 2019; Harris et al., 2015). Children with DCD have difficulties coordinating their movements, which greatly impacts their participation in many aspects of everyday life, like school, self-care, and sports (Reynolds et al., 2024; Zwicker et al., 2013). Despite the high prevalence and large impact, not many people have heard of DCD, or are aware of its existence. This is not only true for society in general, but also for professionals working in health care and education (Meachon et al., 2023). This is striking, given that these professionals are paramount for early diagnosis and intervention, which in turn can decrease the negative impact of DCD. In the current study, we will explore the awareness of DCD among educational professionals in the Dutch primary school system, explore ways to improve this awareness, and identify the role that schools and teachers can play in minimizing the negative outcomes of DCD.

Several studies to date have investigated the awareness and knowledge of DCD among different professional (Hunt et al., 2020; Meachon et al., 2023; Wilson et al., 2012). For example, Meachon et al. (2023) most recently conducted a large-scale survey study across multiple countries and clinical disciplines (e.g., psychologists, physical therapists, etc.), with only 58% of participants reporting awareness of DCD. In addition, only 35.5% of the participants correctly identified a possible DCD diagnoses based on a hypothetic case description. When children are diagnosed for movement difficulties, many different terms are used. In Australia, for example, the majority of children are diagnosed with dyspraxia, which might hinder receiving optimal support (Licari et al., 2021). These numbers are especially striking given that the term DCD has been the official international term for this condition since 1994 (Blank et al., 2019; Polatajko et al., 1995), and has been in the DSM as such from 2013 onwards (American Psychiatric Association, 2013). Nevertheless, the percentage of awareness is similar in studies among professionals in clinical disciplines (Hunt et al., 2020), and even lower among educational professionals (Hunt et al., 2020; Wilson et al., 2012) and parents (Wilson et al., 2012). Teachers have mentioned a lack of professional training opportunities as one of the reasons for this lack of awareness, and highlighted that a correct diagnosis is critical in providing the correct support (Hunt et al., 2020). Even when teachers reported to be aware of DCD, the majority did not recognize the non-motor consequences of the disorder (Wilson et al., 2012). Although these survey studies provide valuable insight into the amount of awareness of DCD, they cannot uncover where and when this awareness originates, nor does it include the view of the professionals on their (un)awareness.

The general unawareness of DCD can negatively impact a timely diagnosis and intervention. In order to prevent negative health outcomes in children with DCD, early identification and diagnosis is paramount (Blank et al., 2019; Missiuna et al., 2008). Currently, children with DCD in the Netherlands are most often diagnosed around the age of 7, with parents and teachers being the first link in the chain leading to a diagnosis (Lust et al., 2022a). This diagnostic trajectory can be quite stressful for families and often take a long time (Ahern, 2000; Alonso Soriano et al., 2015; Klein et al., 2023; Lust et al., 2022a; Missiuna et al., 2006). During this process parents often encounter teachers and professionals that fail to correctly recognize the difficulties and provide the right support (also see Hunt et al., 2020 and Wilson et al., 2012), leading to feelings of frustration and being unheard (Ahern, 2000; Alonso Soriano et al., 2015; Klein et al., 2023). The low level of knowledgeability among teachers and health care providers increases these negative feelings. Given the important role of teachers in the diagnostic process and the especially low awareness previously reported among this group (Hunt et al., 2020; Wilson et al., 2012) this study explores awareness in the primary school system and ways to improve this.

Teachers that are aware of DCD cannot only signal the difficulties that children encounter, but can also support children and lower the (perceived) negative outcomes of DCD. Children with DCD have an increased risk of both physical (Cairney et al., 2019; Zacks et al., 2021) and mental health problems (Green et al., 2006; Lingam et al., 2012). According to the elaborated environmental stress hypothesis both personal (e.g., feelings of mastery, self-esteem) and social resources (e.g., understanding, friendships) influence the consequences of decreased motor skills and negative events like interpersonal conflicts on this physical and mental health (Cairney et al., 2013; Mancini et al., 2016; Mancini et al., 2019). Teachers can affect both types of resources by providing social support (both from themselves and the classmates) and by stimulating the personal resources (e.g., boosting confidence or providing practical support to enhance feelings of mastery) (Jasmin et al., 2014). Parents have reported that both the diagnosis of DCD and the knowledgeability of teachers was related to better support at school (Lust et al., 2022a). In addition, schools can serve as a “vehicle” for intervention and partnerships between education, health services and families as shown by the Partnering for Change project (Missiuna et al., 2012). Parents have also expressed a need for more specific services and therapy at school (Jasmin et al., 2014). As such, the impact of awareness of DCD among the whole school system on the lives of children with DCD should not be underestimated.

In this interview study we aim to give insight into the awareness about DCD in the Dutch primary school system. While several educational concepts exist in the Netherlands (e.g., Montessori schools, or Dalton schools), we focused on both public schools and schools with a catholic background “learning year class system” which represents the majority of the primary schools in the Netherlands. In order to enrich the information, interviews are not only conducted with teachers, but also with directors, special needs coordinators (SNCs) and students from teachers’ colleges for primary education. We specifically included college students to gain insights into differences between the current and next generation of primary school teachers and include any possible changes in the curriculum of aspiring teachers. In addition to the general awareness, the interviews also focused on ways to improve awareness and the support that teachers can (or should) provide. Based on previous studies, we expect awareness to be low, but that the description of DCD is recognizable for (aspiring) professionals. Finally, we expect that these stakeholders in the school system do know how to provide support and increase awareness for DCD.

## 2 Materials and methods

### 2.1 Recruitment

A convenience sample of 21 participants, including students from teachers’ colleges for primary education and professionals in education (between 1 and 35 years of experience working in primary education) participated in the interviews. Recruitment started in the individual networks of two of the researchers (MB, LH) after which the network of the participants was used to recruit additional participants. Recruitment took place between March and April 2024. This procedure led to the inclusion of 10 primary school teachers (8 classroom teachers and 2 physical education (PE) teachers), 7 college students (from teachers’ college for primary education and/or physical education), and 4 other stakeholders in the primary school organization (including 2 school organization directors and 2 SNCs). Participants were between 22 and 57 years old (M = 34.2, SD = 12.5), nine participants did not provide their age. The majority of participants were female (76.2%). Characteristic of the individual participants are shown in Table 1. Teachers, directors and SNCs worked at different independent primary schools, which were part of one school network. The college students followed their education at six different colleges throughout the Netherlands. All participants provided written and spoken informed consent. Ethical approval was provided by the Ethics Committee of the Faculty of Social Sciences of the Radboud University (ECSW-LT-2024-4-4-97240).

**Table 1:** Overview of participants.

Respondent	Gender	Age	Occupation
R1	Female	26	Teacher
R2	Male	28	PE teacher
R3	Male		PE teacher
R4	Female	27	Teacher
R5	Female	57	Teacher
R6	Female	22	Teacher
R7	Female		Teacher
R8	Female	47	Teacher
R9	Female	52	Teacher
R10	Female		Teacher
R11	Female		College student
R12	Male		College student
R13	Female		College student
R14	Female	22	College student
R15	Female	25	College student
R16	Male	21	College student
R17	Female		College student
R18	Male	46	Director
R19	Female	37	Director
R20	Female		Guidance counselor
R21	Female		Special Needs Coordinator

### 2.2 Materials

For this study, an interview guide was developed (see Supplementary Appendix A). The guideline differs slightly between different respondent groups (i.e., teacher, student, or other stakeholder in the school system) and the awareness of DCD of the participant. The interview guide was developed as part of the master thesis of two of the authors, and was extended for that aim with additional questions regarding the overall diagnostic trajectory in primary schools and experience with other types of psychological and physical disorders in the classroom. Nevertheless, the order of the topics was consistent across participants, with all questions relevant for the current research aim preceding the other topics. The questions were developed based on the current research questions and, in for the questions regarding social support, informed by the elaborated environmental stress hypothesis (Cairney et al., 2013).

### 2.3 Procedure

In this study, a semi-structured interview approach was used to collect qualitative data from the participants. This type of interview structures the topics that are being discussed, while also providing enough room for the participant to elaborate on the aspects that are deemed important (Wolgemuth et al., 2014). Interviews were conducted by two researchers (MB, LH), the first interview the researchers conducted together after which the remaining interview were divided between them. All interviews were recorded using a voice recorder app on a mobile phone and had a total duration between 30 and 60 min.

### 2.4 Positionality statement

Data was collected by MB and LH, both master students of pedagogical sciences at the moment of the interviews. They were unaware of DCD before the start of this project. Their knowledge of DCD at the time of the interviews followed a brief literature study, which resulted in an open attitude and genuine curiosity in their questions and respondents’ answers. A risk of the lack of personal experience with DCD is that they might miss some less known signals of DCD in the classroom when teachers explain their experiences.

FvA and HB are assistant professors at the department of pedagogical science. FA has a background in Human Movement Science and has published some papers on DCD. HB has a background in Sociology and also has a research interest in DCD. Both focus on highlighting the problem of unawareness of DCD and ways to improve awareness. By not being present at the interviews, they were able to read the transcripts with an open mind.

### 2.5 Data analysis

All interview recordings were transcribed verbatim, after which they were anonymized by removing names. Transcripts were analyzed by means of thematic analysis, suitable for the exploratory nature of the study (Braun and Clarke, 2006; Braun and Clarke, 2014). This was done in the following steps. First, transcripts were split into units, segments, sentences or parts thereof related to a single statement or topic. Next, the units of the first transcript received a “code.” For this, two researchers (MB, LH) analyzed the transcript individually after which the codes were discussed. This led to the development of a codebook, which was then used to code the remaining transcripts. To identify emerging patterns and themes, the codes were further discussed, similar codes were grouped into subcategories and then into themes. To determine inter-coder reliability, a third researcher (FA) coded three random transcripts, showing good reliability (Cohen’s kappa = 0.83). The third researcher then checked the remainder of the coding scheme and made small adjustments to better suit the aims of this paper. The complete coding scheme can be found in Supplementary Appendix B.

## 3 Results

### 3.1 Awareness, diagnosis, importance

**Figure 1:**
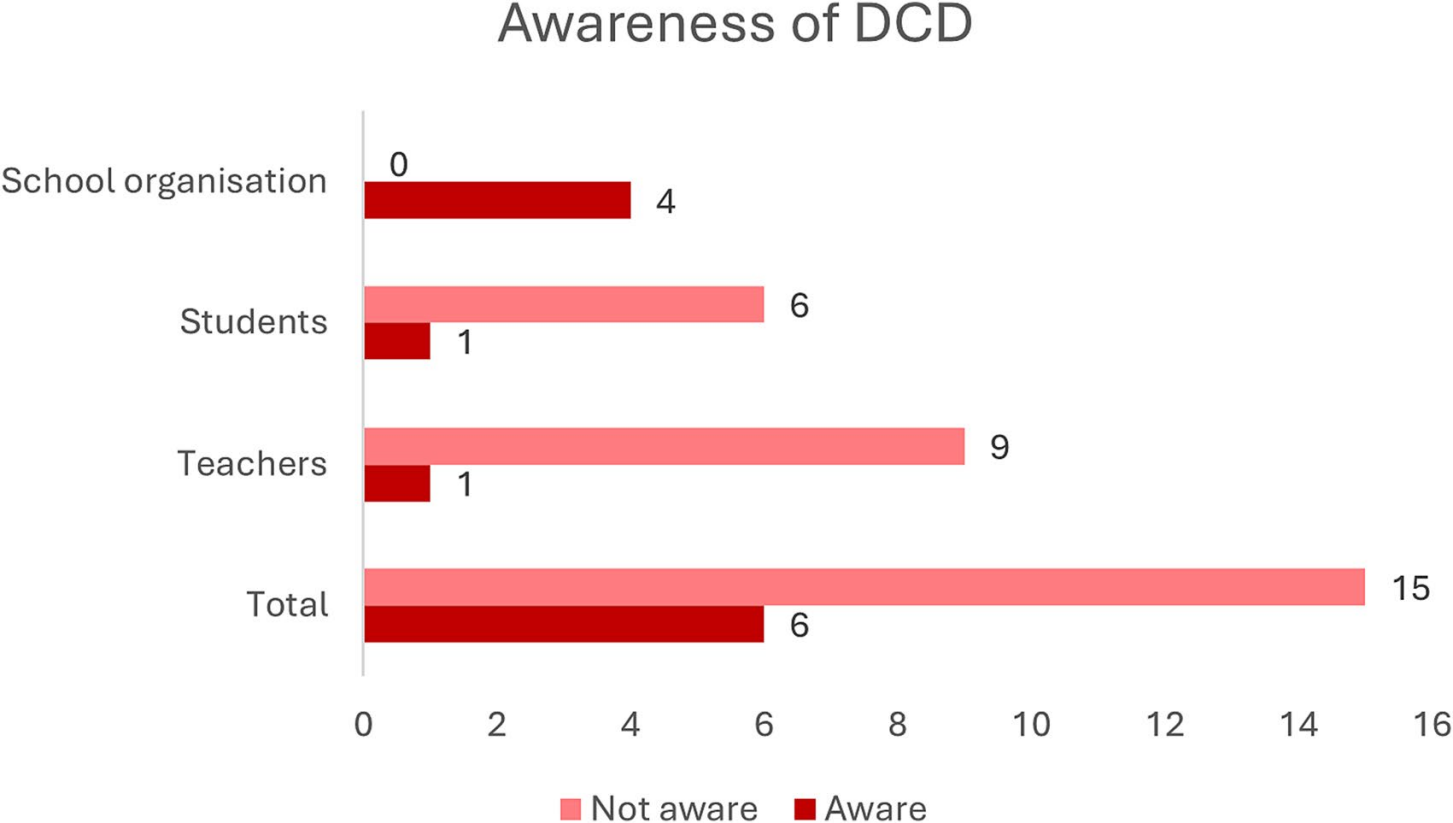
Number of respondents aware of DCD.

Results showed that only 28.6% (6/21 respondents) of the participants were aware of the existence of DCD (see Figure 1). This number differed largely between participant groups, with the highest percentage among participants from the school organization (4/4 respondents), followed by college students (1/7 respondents), and teachers (1/10 respondents). One participant received information in extra-curricular education, three participants encountered a child with DCD in the work context and two participants know a child with DCD in a personal setting. After receiving information about DCD by means of a poster with often occurring difficulties and behaviors of children with DCD, 14 out of the 15 participants without awareness recognized the symptoms. “*Yes of course when you think about it, that’s the funny thing, then you think back to children and then you sometimes think that child was maybe a bit like that. But not so that there was a label on it”* (R7).

All participants endorse the importance of increasing awareness and the relevance of diagnosing this condition. They mention both reasons from the perspective of the child (e.g., better understanding, acceptance) as well as from a professionals perspective (e.g., providing appropriate support, receiving input from specialists). *“If you have knowledge about DCD then you also know what the consequences are and what you can do to prevent these consequences”* (R20).

The role of the teacher in the diagnostic trajectory is primarily seen as signaling, but also communicating their concerns with parents, SNCs, and PE teachers is mentioned. “*the teacher needs to observe, then you notice if anything is wrong with a child or not. Then you can engage in a conversation with the parents, or a fellow teacher for multiple viewpoints”* (R3).

### 3.2 Personal and social support

**Figure 2:**
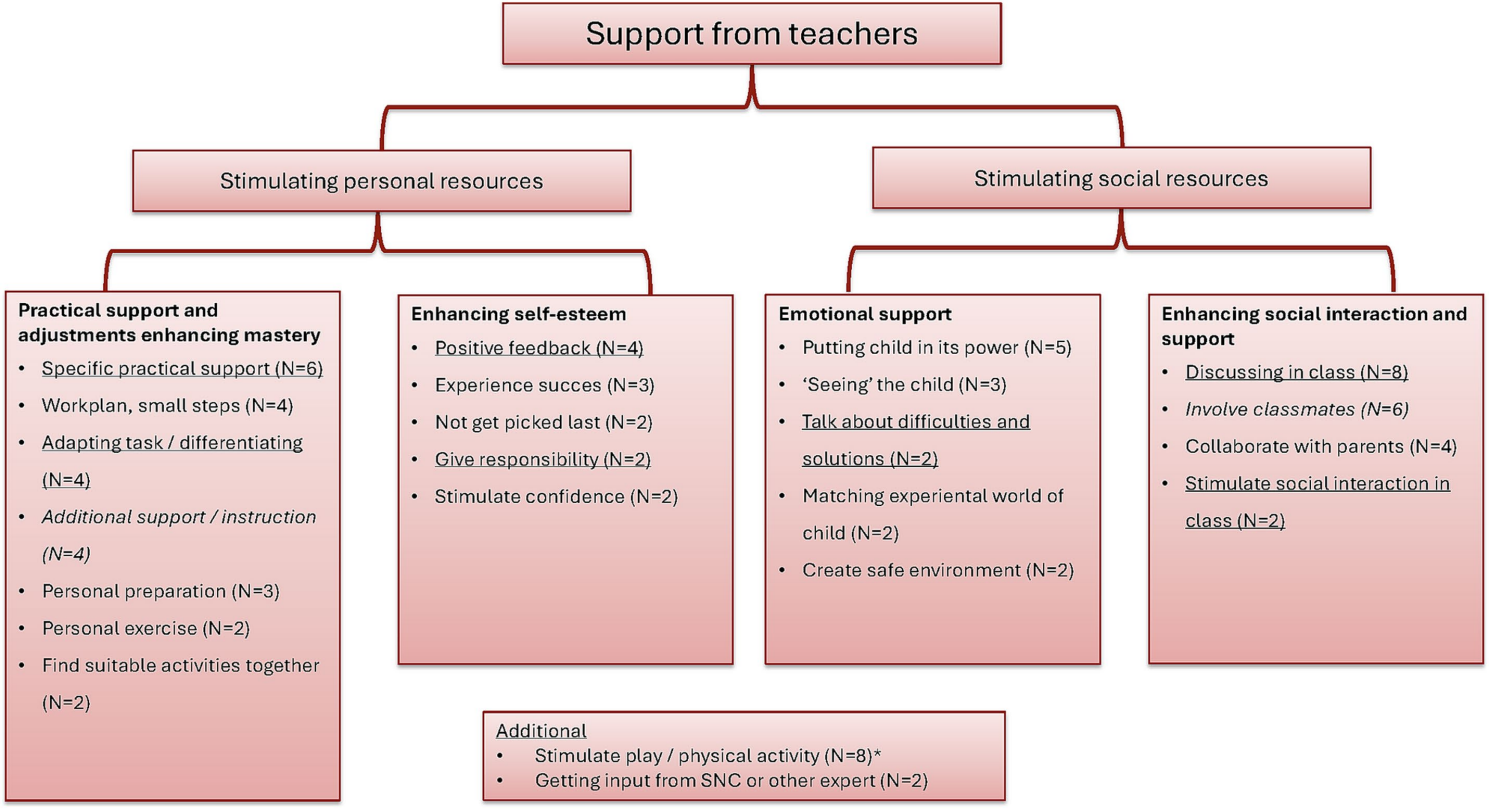
Suggested support provided by teachers for children with DCD. The figure includes responses mentioned more than once. In addition to these responses, 11 forms of support were mentioned by individual respondents. Underlined responses are primarily given by teachers (≥75%), responses in italics are primarily given by college students (≥75%). *Indicates response also given by a respondent from the school organization.

Even though general awareness was low, participants were able to come up with multiple ways in which they can support children with DCD. In total, 83 responses (49 from teachers, 34 from college students) related to a form of support, of which 44 (24 from teachers, 20 from college students) focused on personal resources and 39 (25 from teachers, 14 from college students) focused on social resources (see Figure 2). To stimulate personal resources, the majority of responses related to practical support and enhancing mastery. Several aspects stand out here. Interestingly, the specific practical support was only mentioned by teachers and not by college students. This type of support included, for example, structuring a drawer of providing special paper or pens to help with writing. “*Or they get larger lines, wider lines, to make it easier, to make it more readable”* (R5). The college students did mention practical support, but in more general descriptions of providing additional support or additional instructions (4/4 responses from college students). “*Because I think it might be nice to get some extra attention in that case”* (R14). With regards to social resources, most responses related to enhancing social interaction. For this goal, teachers and college students both mention to discuss the topic in class. In addition, the college students mentioned to really involve classmates to improve participation of a child with DCD during the school day (5/6 responses from college students). *“And maybe you think about: where do I put this child within the classroom, next to another pupil that can help him or her with some things so to say”* (R16). A final noteworthy finding is the mentioning of stimulating play or physical activity. It needs to be noted here that participants were prompted during the interview on the possible physical consequences of DCD, including inactivity and risk of obesity. “*… then I think a child could be helped despite the challenges he experiences in moving. Still start to enjoy physical activity. I think that is very important”* (R18).

In addition to the support from teachers, support can also be provided on the level of the school organization (see Table 2 for suggestions made more than once). This form of support focusses primarily on a stimulating environment for children and teachers, and enhancing collaborations between professionals. It stands out that the teachers are primarily focused on the awareness in the school. “*Well, I think it would definitely help if there would be more knowledge school-wide. And you can do that for example with posters in the teacher lounge, or talking about it during a meeting, or writing a protocol”* (R6). On the other hand, college students mention specific aspects of additional support that could be provided within the school organization. *“… extra support. That they get once a week, uhm, some sort of tutoring. Maybe if you have a teaching assistant at school for that*” (R11).

**Table 2:** Support that can be provided by the school system.

	Teachers	College students	School organization
Create awareness in school	5	1	1
Extra support (assistants, experts)	1	4	–
Simulate safe and supportive climate	–	2	2
Projects for group dynamics	1	–	1
More (collaboration with or attention for) PE	1	2	–
External collaborations	1	1	2

### 3.3 Improving awareness

**Figure 3:**
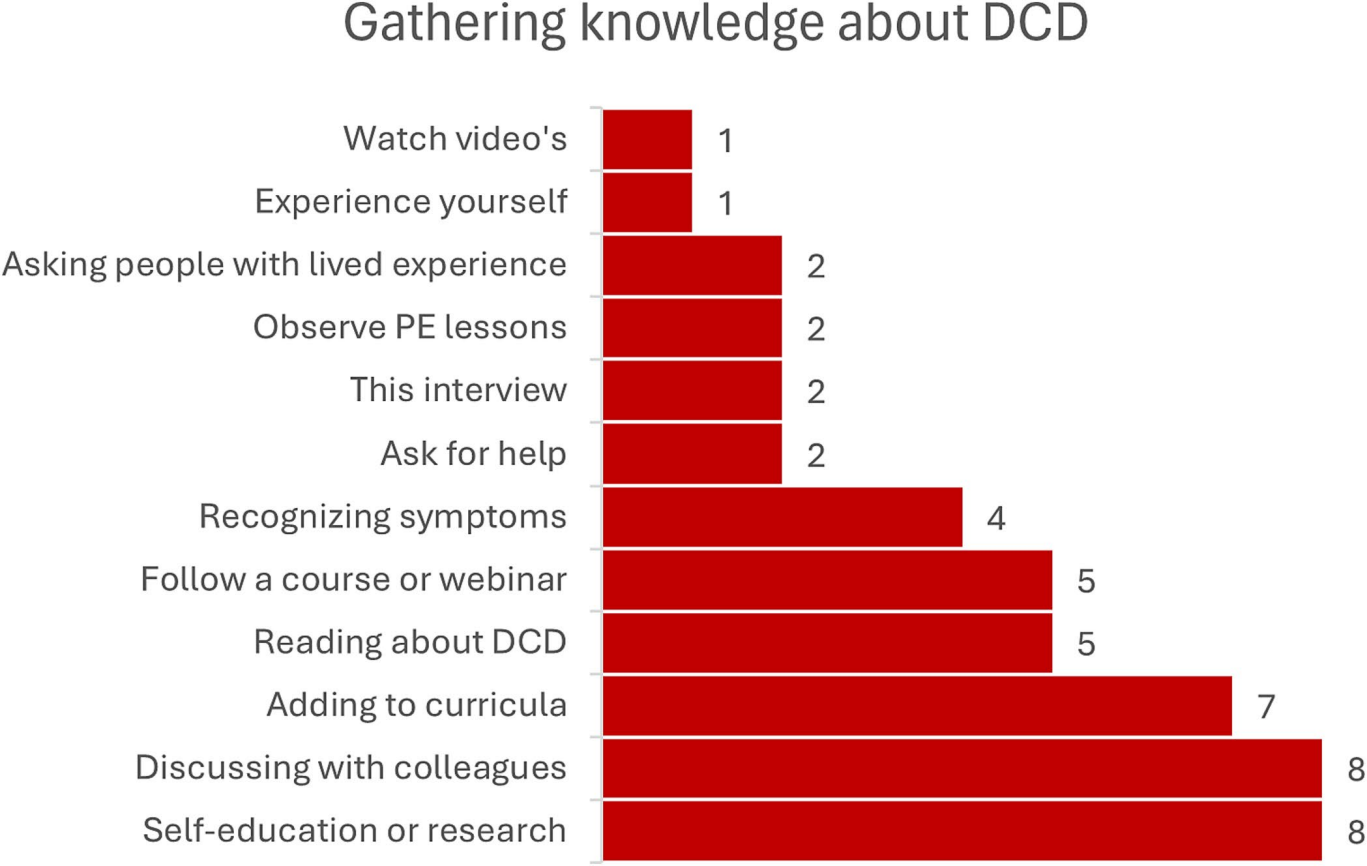
Ways in which respondents can increase their awareness of DCD.

For improving awareness, we first asked participants what they can do themselves. All responses can be seen in Figure 3. Most responses relate to self-education and discussing the topic with colleagues. It is also noteworthy that all college students were open to the idea that DCD should be part of the curriculum, both for primary school teachers and for PE teachers.

**Figure 4:**
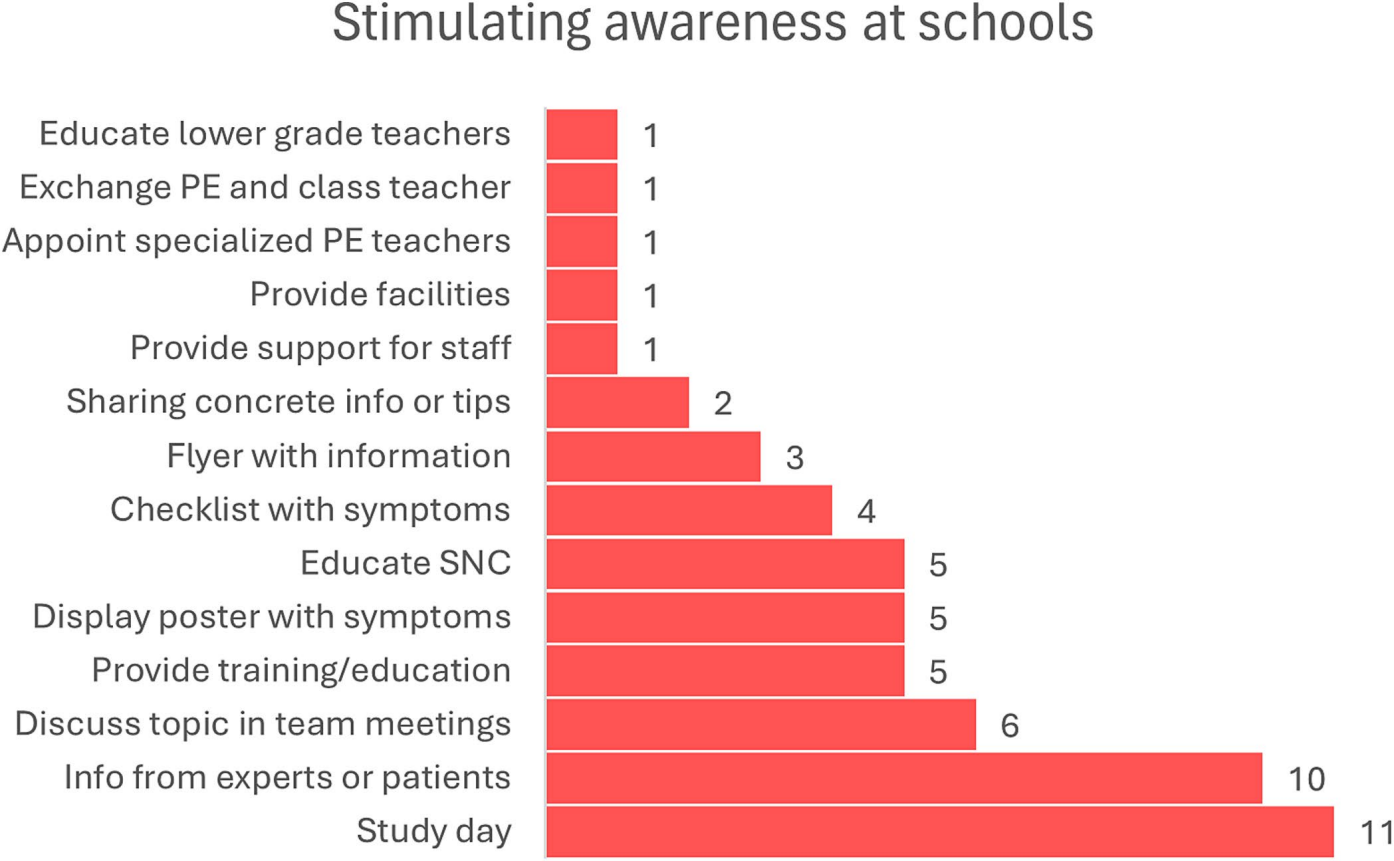
Ways to increase awareness of DCD in the school system.

We then asked what can be done or provided by the school system to raise awareness on a school-level. Many different options were mentioned (see Figure 4), but all included that information needs to be hands on and should be easy to apply the next day. “…*make a kind of checklist of how to help a child with dcd. How can I tell if someone has it or not*” (R2). They also highlighted that teachers cannot be up to date on all possible disorders they might encounter, which is why they specifically mentioned the role of the SNC.

Finally, we asked about the responsibility for raising awareness of DCD in the school system (see Table 3). The responses indicate that the initiative of raising awareness lies primarily with the SNC and school administration.

**Table 3:** Stakeholder responsible to raise awareness of DCD.

	Teachers	College students	School organization
Special needs coordinator	3	3	3
School administration	3	4	1
Specialists	3	1	1
Patient organization	1	1	1
Teachers	–	2	–
Whole team at school	1	–	–
School partnerships	1	–	–

## 4 Discussion

This study set out to explore the awareness of DCD from the perspective of stakeholders in the Dutch primary school system, ways to improve awareness, and social support provided by teachers. Despite the low awareness, (aspiring) professionals are open to ways to improve awareness and recognize the importance. They also acknowledge the important role they can play in supporting children with DCD, in their diagnostic trajectory and in the classroom, and in raising awareness.

The low awareness of teachers in primary education is in line with previous large-scale survey studies (Hunt et al., 2020; Wilson et al., 2012). It highlights that even though the need to increase awareness is clear, there is still a long way to go (Steenbergen et al., 2024). We extended previous findings by showing that within the school system this situation is not yet likely to change as indicated by the low awareness among college students and the absence of information about DCD in the curricula (which is primarily focused on didactics and pedagogics). Given that parents often worry about problems related to school and education, and describe a lack of knowledge and support at school (Lust et al., 2022a; Reynolds et al., 2024), this is a troubling prospect. Nevertheless, the symptoms of DCD are very recognizable in practice, which would be expected given the high prevalence. Teachers are aware of the signaling function they can play in the diagnostic trajectory and confirm the probable benefits of improved awareness for both the diagnosis and the support they can provide. This is in line with the relief and acknowledgement which parents report after receiving a DCD diagnosis for their child (Ahern, 2000; Alonso Soriano et al., 2015).

Despite the low awareness, (aspiring) teachers were capable of formulating the support they would provide, which might strengthen both the personal and social resources of children with DCD. From the perspective of personal resources, stimulating (feelings of) mastery and self-esteem can support children in everyday tasks (Cairney et al., 2013; Skinner and Piek, 2001) and matches the needs previously identified by parents in the context of school (Klein et al., 2023, Jasmin et al., 2014). In addition, the strengthening of social resources may lighten the difficulties with socialization, which was the most often reported difficulty in the school context in Australia (Reynolds et al., 2024). This support is in line with support provided by school reported in a previous study (Reynolds et al., 2024). It needs to be noted here that in some cases respondents used examples of support that can also be more generally applicable to neurodivergent children, which was sometimes explicitly stated by participants. Nevertheless, they do fit the identified needs and are often in line with existing interventions and programs promoted by advocacy groups and parent/patient organizations like Balans (2025). Apart from the support fitting within the context of the environmental stress hypothesis, it is interesting to notice that teachers also mention stimulating play and physical activity. Even though information provided during the interview might have triggered this response, it is promising given that children with DCD often participate less in physical activity and have an increased risk of obesity (Cairney et al., 2019; Zacks et al., 2021). Children with DCD often lack enjoyment in participating in physical activity (Reynolds et al., 2024; Tamplain et al., 2024), so more emphasis on this aspect in a school setting is warranted. Together, the findings show that professionals in the educational system are well equipped to provide support when being aware to do so.

To stimulate awareness of DCD and increase the opportunities for children to receive the most effective support, advocacy groups and patient organizations aim to spread freely available resources. In the Netherlands, vereniging Balans shares information for parents as well as for professionals in education.^1^ These efforts are similar to Developmental Coordination Disorder Australia Inc. (2025) and CanChild (2025) in Australia and North-America, respectively. But despite all these efforts, awareness still remains low (Steenbergen et al., 2024). To our knowledge, no systematic research has been done on the effectiveness of the advocacy efforts, which might prove to be a valuable next step. There are a few exceptions. For example, Camden et al. (2015) showed that an online module can enhance knowledge about DCD and skills regarding evidence-based practice for these children among physical therapists. Our results show that all stakeholders in the educational system are very open to receiving more information about DCD, especially hand-on tips and tools. It might be even more effective to start bottom-up by including DCD in the curriculum for training teachers and SNCs. Educational policy in the Netherlands aims for inclusive education, indicating that every child (also with a disability) should be able to receive education at regular schools in their neighborhood with the right support (Government of the Netherlands, 2025).^2^ However, this support is primarily focused on the more “visible” disorders (e.g., children dependent on a wheelchair or walking aid) and the more well-known disorders (e.g., ADHD, ASD). Our results already highlighted that teachers cannot be knowledgeable about all disorders, which leads to the suggestion that efforts could be directed to the additional support that can be provided at schools.

While the results of the study provide important insights into the (possible) support of the school system for children with DCD, some limitations need to be noted. First of all, awareness of DCD was low. As a result, participants received a short introduction and a poster with the most occurring difficulties of children with DCD in a school context. Even though this strategy proved to be effective to continue the conversation, the provided responses are based on this information. This information does not include the complete complexity and variety in presentations of DCD (Lust et al., 2022b; Schoemaker et al., 2024), which may have narrowed the responses. Nevertheless, given the depth of the conversations, we are confident that the responses cover the most prominent aspects of social support and promoting awareness. Next, the small sample size, especially among the participants in the school organization, limits the generalizability of the findings. This is especially true for the awareness found in this group, as anecdotal evidence shows that definitely not all school directors and SNCs are aware of DCD. In addition, all teachers, directors and SNCs were part of the same school network, even though they do work at independent schools, and represent only a small part of the primary schools in the Netherlands, which further lowers the generalizability of the findings. On the other hand, the participants in the college student group did follow their education in six different colleges. Therefore, findings like the lack of attention in the curriculum do reflect the current state in the Netherlands.

## 5 Conclusion

We showed that awareness of DCD, from the perspective of our participants within the Dutch primary school system, is low. A remarkable outcome is that awareness is lowest among the group that interacts with the children on a daily basis, that is the teachers. Nevertheless, the different stakeholders are willing to change this. While responsibility is primarily placed at school directorates or special needs coordinators, (aspiring) teachers also acknowledge their role in signaling difficulties and providing the right support. Awareness should be increased within society as a whole to alleviate the difficulties of children with DCD, and the school system might provide a suitable starting point.

## Data Availability

The raw data supporting the conclusions of this article will be made available by the authors, without undue reservation.

## Footnotes

^1^ https://balansdigitaal.nl/ondersteuning-bij/dcd

^2^ https://www.government.nl/topics/primary-education/appropriate-education-at-primary-school
