# Supplementary material for: Awareness of DCD from the perspective of stakeholders in the Dutch school system: a qualitative study
Source: Front Hum Neurosci. 2025 Jun 23;19:1593912. doi: 10.3389/fnhum.2025.1593912 (PMC12230003; doi:10.3389/fnhum.2025.1593912)
Supplement: Supplementary file 1 [file Supplementary_file_1.docx]

**Appendix A. Interview guide**

Purpose of Interview:

For our thesis, we are conducting research on the disorder DCD and its awareness. With this interview, we want to gain insight into what the current awareness is among involved elementary school students

Background Variables

- Who are you?

- What is your job title/what education are you pursuing?

For participants unaware of DCD

Note: for participants unaware of DCD, the description of the topics were similar for the different stakeholder groups, but differed slightly between the overarching topics of awareness, psychological consequences and physical consequences.

*Awareness*

- Awareness of DCD

- Make link with other disorder such as ADHD (to raise awareness), show poster → question their knowledge

- Point out the importance of DCD

- Solicit ideas to increase awareness

*Psychological consequences*

- Give introduction about psychological consequences

- Solicit ideas to increase awareness of psychological consequences

*Physical consequences*

- Provide introduction on physical consequences

- Ideas to elicit more awareness of physical consequences

For participants aware of DCD

Note: for participants who are aware of DCD, the description of the topics was similar for awareness, psychological consequences and physical consequences, but differed slightly between stakeholder groups.

*For teachers:*

- Awareness about DCD (or psychological/physical consequences).

- Experiences in the classroom/diagnosis

- Ideas to increase awareness of DCD.

*For school organization*:

- Awareness regarding DCD (or psychological/physical consequences)

- Experiences in school / diagnosis

- What are you doing as directors to increase awareness of DCD (and psychological/physical consequences)?

- Ideas to increase awareness

*For students*:

- Awareness about DCD (or psychological/physical consequences)

- Attention to DCD within the curriculum

- Experiences with internship / diagnosis

**Appendix B. Coding scheme**

| Teachers | | | | |
| --- | --- | --- | --- | --- |
| Theme | **subject** | **Sub-subject** | **Code** | **Respondents** |
| Awareness | Knowledge of DCD | Yes or no | Yes | **R8** |
|  |  |  | No | R1, R2, R3, R4, R5, R6, R7, R9, R10 |
|  |  | Reason of awareness | Private situation |  |
|  |  |  | Work/student | **R8** |
|  |  |  | Education |  |
|  | Recognising symptoms |  | Recognition | R1, R2, R3, R5, R6, R7, R9, R10 |
|  |  |  | No recognition |  |
|  |  |  | Just a bit | R4 |
|  | Importance of awareness and diagnosis | For child | Explanation | R1, R5, R7, R9 |
|  |  |  | Feeling seen and understood/relief | R3, R4,R6, R7, **R8** |
|  |  |  | Understanding for themselves / acceptance | R1, R4, R5 |
|  |  |  | Prevalence | R6, R5, R10 |
|  |  |  | Motivating/confidence | R7 |
|  |  |  | Get right support/have additional value | R9, R10 |
|  |  |  | Invisible nature | R6, R7 **R8** |
|  |  | For teacher | Optimal support & environment | R1, R2, R3, R4, R5, R10 **R8** |
|  |  |  | Conversation input | R1 |
|  |  |  | Attentiveness/signaling | R1, R3, R7 |
|  |  |  | Prevention | R3, R4, R5, R7, R9 **R8** |
|  |  |  | Patience | R1 |
|  |  |  | Cause of behaviors/issues | R5 |
|  |  |  | Take condition into account | R6 |
|  |  |  | Get practical support | R10 |
|  |  |  | Understanding child | **R8** |
|  |  |  | No additional value, not keen on diagnoses | R1, R4, R6, **R8** |
|  | Own ideas about disorder and its consequences | General | Developmental problems | R6 |
|  |  | Psychological | Unhappy | R1 |
|  |  |  | Insecure | R1, R2, R3, R4, R10 |
|  |  |  | Social issues | R2, R5 |
|  |  |  | Bullying | R3 |
|  |  |  | loneliness | R4, R5 |
|  |  |  | Misunderstood | R4 |
|  |  |  | No/few hobbys | R5 |
|  |  |  | Low self-esteem, feelings of inferiority | R6, R7, R9, R10 |
|  |  |  | depression | **R8** |
|  |  |  | Burn-out | **R8** |
|  |  | Physical | Headache | R1 |
|  |  |  | Cramped muscles | R1 |
|  |  |  | Motor activity issues (prevalent) | R2, R3, R5, R6, R7, R9 |
|  |  |  | Influence on growth | R4 |
|  |  |  | Overweight | R6, R7 |
|  |  |  | Dislike activities | R9 |
|  |  |  | Accidents/pain | R10 |
| Social support | From teacher |  | Practical support | R1, R5, R6, R7, R10, **R8** |
|  |  |  | More time | R1 |
|  |  |  | Personal preparation | R1, R6 |
|  |  |  | Discussing in class | R1, R4, R5, R9, R10 **R8** |
|  |  |  | Not get picked last | R1 |
|  |  |  | Differentiating or adapting task | R2, R5, R8 |
|  |  |  | Small steps, working stepwise | R2, R6 |
|  |  |  | Give responsibility in activities | R3, **R8** |
|  |  |  | Talking with kid about difficulties and solutions | R1, R3 |
|  |  |  | Positive feedback | R2, R9 **R8** |
|  |  |  | Understanding | R2, |
|  |  |  | Putting child in its power | R2, R5, R7 |
|  |  |  | Involving in class | R3, |
|  |  |  | Specific/personal exercise | R4 |
|  |  |  | Letting child explain in class | R4 |
|  |  |  | Finding suitable activities together | R4 |
|  |  |  | Collaborate with parents | R5, R9 |
|  |  |  | Stimulating social interaction | R5, R7 |
|  |  |  | ‘seeing’ the child | R9 **R8** |
|  |  |  | Stimulate play/physical activity | R7, R9, R10 |
|  |  |  | Experience success | R7 |
|  |  |  | Protecting | R7 |
|  |  |  | Experiential world of child | R7 |
|  |  |  | Getting input from SNC | R9 |
|  |  |  | Create save environment | **R8** |
|  |  |  | Setting boundaries | **R8** |
|  |  |  | Involve classmates | **R8** |
|  | From school system |  | Creating awareness/understanding | R1, R4, R6, R9 **R8** |
|  |  |  | Extra support education-assistants | R3 |
|  |  |  | Organise meetings with parents | R4 |
|  |  |  | Collaboration PE and regular teacher | R5 |
|  |  |  | Schoolwide projects groupdynamics | R5 |
|  |  |  | Sharing experiences | R6 |
|  |  |  | Early signalling | R7 |
|  |  |  | External collaborations | R10 |
| Stimulating awareness | Role teacher | Gathering knowledge | This interview | R1 |
|  |  |  | Self-education/research | R1, R3, R4 |
|  |  |  | Reading/book | R1, R2, R4, R10 |
|  |  |  | Discussing | R2, R5, R6, R7, R10 |
|  |  |  | Course or webinar | R2, R4, R9, R10 |
|  |  |  | Asking for help | R5 |
|  |  |  | Recognizing symptoms | R5 |
|  |  |  | Not all teachers | **R8** |
|  |  | Sharing knowledge | Talking about interview | R3, R9 |
|  |  |  | Discussing with parents | R5 |
|  |  |  | Talking about it | R10 **R8** |
|  | Role school system | Gathering knowledge |  |  |
|  |  | Sharing knowledge | Expert information | R1, R2, R3, R4, R5, R6 |
|  |  |  | Discussing topic/meetings | R1, R2, R5, R6 |
|  |  |  | Receiving info, tips | R1, R2 |
|  |  |  | Study day | R1, R3, R4, R5 |
|  |  |  | Checklist | R2, R3 |
|  |  |  | Flyer | R2,R4 |
|  |  |  | Training/education | R4, R6, R7 |
|  |  |  | Poster with symptoms | R5, R6, R7 |
|  |  |  | Educating SNC | R9, R10 **R8** |
|  |  |  | Provide support for staff | **R8** |
|  |  |  | Getting right facilities | **R8** |
|  | Responsibility |  | SNC | R1, R2, **R8** |
|  |  |  | Behaviour specialist | R1 |
|  |  |  | Management | R4, R5, R6 |
|  |  |  | Whole team | R5 |
|  |  |  | Balans |  |
|  |  |  | School partnerships | R7 |
|  |  |  | Professionals (therapists) | R9 |
|  |  |  | Umbrella organisation | R10 |
|  | Role education |  | ALO | R2 |
|  |  |  | PABO | R6, R7, R10 |
|  | General |  | Database/knowledgebase | R3 |
|  |  |  | Tracking system for children | R2, R3 |
| Diagnosis trajectory | Role teacher |  | Talking with parents | R1, R3, R6 |
|  |  |  | Signalling | R2, R3, R4, R5, R6, R7, R9, R10, **R8** |
|  |  |  | Involve expert | R3 |
|  |  |  | Talking with people involved in diagnosis | R4 |
|  |  |  | Talking with kid | R5 |
|  |  |  | Follow protocols | R6 |
|  |  |  | Support investigation | R9 |

| Students | | | | |
| --- | --- | --- | --- | --- |
| Theme | **subject** | **Sub-subject** | **Code** | **Respondents** |
| Awareness | Knowledge of DCD | Yes or no | Yes | **R13** |
|  |  |  | No | R11, R12, R14, R15, R16, R17 |
|  |  |  | Education | **R13** |
|  | Recognising symptoms |  | Recognition | R12, **R13,** R14, R15, R17 |
|  |  |  | No recognition |  |
|  |  |  | Just a bit | R11, R16 |
|  | Importance of awareness or diagnosis | For child | Explanation | R16 |
|  |  |  | Prevalence | R12, **R13,** R14, R16 |
|  |  |  | No awareness | R11, R12 |
|  |  |  | Prevent later issues | R14 |
|  |  | For teacher | Optimal support & environment | R12, **R13,** R14, R15, R16 |
|  |  |  | Attentiveness/ early signalling | R14 |
|  |  |  | Prevention | R11, R12, R14, R15, R17 |
|  |  |  | Take into account | R16 |
|  |  |  | Understanding child | R16 |
|  |  |  | Recognizable | R14 |
|  | Own ideas about disorder and its consequences | General | Developmental problems |  |
|  |  |  | Disorder in coordination | R11, R17 |
|  |  |  | Deficit/dysfunction | R12, R15 |
|  |  |  | Motor disorders with symptoms | **R13** |
|  |  |  | Cognitive disorder | R14 |
|  |  |  | Delay motor development | R14, R16 |
|  |  | Psychological | Unhappy |  |
|  |  |  | Insecure | **R13,** R15, R16 |
|  |  |  | Rebellious | R15 |
|  |  |  | Low self-esteem, feelings of inferiority | R14, R17 |
|  |  |  | Anxiety | R11, R12 |
|  |  |  | Embarrassment | R11 |
|  |  |  | Negative emotions | R12, **R13,** R14 |
|  |  |  | Fear | R14 |
|  |  |  | Frustration | R15 |
|  |  |  | Exclusion | R17 |
|  |  | Physical | Headache |  |
|  |  |  | Motor activity issues (prevalent) | R11, R14 |
|  |  |  | Overweight | R16 |
|  |  |  | Accidents/pain | R17 |
|  |  |  | (Motor) delay | R12, R15, R16 |
|  |  |  | Fatigue | R15 |
|  |  |  | Illnesses | R1 |
| Social support | From teacher | Stimulating confidence | Personal preparation | R12 |
|  |  |  | Discussing in class | **R13,** R17 |
|  |  |  | Not get picked last/prevent exclusion | R17 |
|  |  |  | Small steps, working stepwise | R11, R12 |
|  |  |  | Stimulate confidence/ prevent insecurities | R15 |
|  |  |  | Positive feedback | R17 |
|  |  |  | Putting child in its power | R14, R15 |
|  |  |  | Specific/personal exercise | R14 |
|  |  |  | Finding suitable activities together | R14 |
|  |  |  | Adapting task | R16 |
|  |  |  | Collaborate with parents | R11, R14 |
|  |  |  | ‘seeing’ the child | R15 |
|  |  |  | Stimulate play/physical activity | **R13,** R14, R15, R17 |
|  |  |  | Experience success | R11, R12 |
|  |  |  | Matching experiental world of child | R11 |
|  |  |  | Getting input from Iber or other expert | R17 |
|  |  |  | Create save environment | **R13** |
|  |  |  | Involve classmates | R11, R12, R14, R15, R16 |
|  |  |  | Extra support/instruction | R11, R14, R15, R16 |
|  |  |  | Structuring | R12 |
|  |  |  | Feelings of competence | R12 |
|  |  |  | Build trust | **R13** |
|  |  |  | Inform other teachers | **R13** |
|  |  |  | Small groups | R15 |
|  |  |  | Prevent pressure | R15 |
|  |  |  | Keeping an eye on | R16 |
|  | From school system |  | Creating awareness/understanding | R17 |
|  |  |  | Extra support (education-assistants, external or internal) | R11, R12, R14, R16 |
|  |  |  | Save environment | **R13,** R15 |
|  |  |  | External collaborations | R14 |
|  |  |  | Offer individual support | R15 |
|  |  |  | Extra classes (PE, Plus) | R15 |
|  |  |  | Practical support | R16 |
|  |  |  | More attention for PE | R17 |
| Stimulating awareness | Role teacher | Gathering knowledge | Self-education/research | R11, **R13,** R14, R15, R17 |
|  |  |  | Reading/book | R16 |
|  |  |  | Asking for help, expert help | R14 |
|  |  |  | Recognizing symptoms | R12, R14, R15 |
|  |  |  | Discussing with colleauges | R11, R15 |
|  |  |  | Observing PE lessons | R12, R16 |
|  |  |  | Experiencing | **R13** |
|  |  |  | Lived experience | R15, R17 |
|  |  |  | Video’s | R15 |
|  |  | Sharing knowledge | Talking about it | **R13,** R14 |
|  | Role school system | Gathering knowledge |  |  |
|  |  | Sharing knowledge | Expert information | R12 |
|  |  |  | Discussing topic/meetings | R12, R17 |
|  |  |  | Study day | R11, R12, **R13,** R14, R17 |
|  |  |  | Checklist | R17 |
|  |  |  | Training/education | R15, R16 |
|  |  |  | Poster with symptoms | R11, R16 |
|  |  |  | Educating SNC | R15 |
|  |  |  | Specialized PE teachers | R14 |
|  | Responsibility |  | SNC | R12, **R13,** R15, R17 |
|  |  |  | Management | R12, **R13,** R14 |
|  |  |  | Whole team | R11 |
|  |  |  | Balans | R16 |
|  |  |  | School partnerships | R16 |
|  |  |  | Teachers | R14 |
|  |  | PABO | Exam committee | R11 |
|  |  |  | Students | R11 |
|  |  |  | Petition | R11 |
|  |  |  | Implement in PABO-ALO | R14, R16 |
| Education | Disorders in curriculum | DCD | Yes |  |
|  |  |  | No | All |
|  | Ideas |  | Yearly lecture | R11 |
|  |  |  | Whole course | **R13** |
|  |  |  | experiencing | **R13** |
|  |  |  | Signalling | **R13** |
|  |  |  | The right support | R15 |
|  |  |  | Cases with examples | R15 |
|  |  |  | general | R12 |
|  |  |  | Teaching from experts | R17 |
| Diagnosis trajectory | Role teacher |  | Talking with parents | **R13,** R14, R15 |
|  |  |  | Signalling | R11, R12, **R13,** R14, R15, R16 |
|  |  |  | Involve expert | **R13,** R14 |
|  |  |  | Referring | R11 |
|  |  |  | Interested & considerate | R11 |

| School organization | | | | |
| --- | --- | --- | --- | --- |
| Theme | subject | Sub-subject | Code | Respondents |
| Awareness | Knowledge of DCD | Yes or no | Yes | R18, R19, R20, R21 |
|  |  |  | No |  |
|  |  | Reason of awareness | Private situation | R18, R20 |
|  |  |  | Work/student | R19, R20, R21 |
|  |  |  | Education | R19 |
|  | Importance of awareness or diagnosis | For child | Getting support | R18, R19 |
|  |  |  | Understanding for themselves | R21 |
|  |  |  | Feeling understood | R20 |
|  |  |  | Support for parents | R19 |
|  |  |  | Recognition | R18 |
|  |  | For teacher | Optimal support & environment | R19, R20, R21 |
|  |  |  | Prevention | R20 |
|  |  |  | Understanding child | R18, R20, R21 |
|  |  |  | Getting practical support | R19 |
|  |  |  | Cause of behaviors/issues | R20, R21 |
|  |  |  | Get to act | R21 |
|  |  |  | No additional value, not keen on diagnoses | R20, R21 |
|  | Own ideas about disorder and its consequences | General | Problems with movement | R21 |
|  |  |  | Developmental disorder motor coordination | R18 |
|  |  |  | Good description of problems in activities | R20 |
|  |  | Psychological | Unhappy |  |
|  |  |  | Insecure | R18, R20 |
|  |  |  | Bullying | R18 |
|  |  |  | Shy | R19 |
|  |  |  | Low self-esteem, feelings of inferiority, negative self-image | R21 |
|  |  |  | Suicide | R21 |
|  |  |  | depression | R19, R21 |
|  |  | Physical | Headache |  |
|  |  |  | Problems ADL | R19, R21 |
|  |  |  | Problems independent living | R19 |
| Social support | From teacher |  | Stimulate enjoyment in play/physical activity | R18 |
|  | From school system |  | Creating awareness/understanding | R20 |
|  |  |  | Stimulate supportive pedagogical climate | R18, R21 |
|  |  |  | Support teachers (as SNC) | R21 |
|  |  |  | Collaboration teachers and other experts | R19 |
|  |  |  | Schoolwide projects groupdynamics (buddy system) | R19 |
|  |  |  | Providing practical support | R20 |
|  |  |  | External collaborations | R20 |
| Stimulating awareness | Role teacher/director | Gathering knowledge | This interview | R18 |
|  |  |  | Webinar | R21 |
|  | Role director |  | Sharing knowledge | R18 |
|  |  |  | Utilizing network | R18 |
|  |  |  | Putting on agenda | R18 |
|  | Role SNC |  | Sharing and gathering knowledge | R21 |
|  |  |  | Smooth transfer between teachers/classes | R21 |
|  |  |  | Discussions | R21 |
|  | Role school system | Gathering knowledge |  |  |
|  |  | Sharing knowledge | Expert information or lived experiences | R20, R21 |
|  |  |  | Study day/presentation | R18, R20 |
|  |  |  | Checklist , signalling chart | R19 |
|  |  |  | Flyer | R20 |
|  |  |  | Discussions PE and regular teacher | R18 |
|  |  |  | Professionalise lower grade teachers | R18 |
|  |  |  | Educating SNC | R20 |
|  |  |  | Online course | R18 |
|  | Responsibility |  | SNC | R18, R19, R21 |
|  |  |  | Management | R18 |
|  |  |  | Balans | R20 |
|  |  |  | Specialists | R20 |
| Diagnosis trajectory | Role teacher |  | Talking with parents | R20 |
|  |  |  | Signalling | R18, R19, R20, R21 |
|  |  |  | Follow-up | R19, R20 |
|  |  |  | Relation with child | R21 |
